# Four‐jointed knock‐out delays renal failure in an ADPKD model with kidney injury

**DOI:** 10.1002/path.5286

**Published:** 2019-06-17

**Authors:** Chiara Formica, Hester Happé, Kimberley AM Veraar, Andrea Vortkamp, Marion Scharpfenecker, Helen McNeill, Dorien JM Peters

**Affiliations:** ^1^ Department of Human Genetics Leiden University Medical Center Leiden The Netherlands; ^2^ Department of Pathology Leiden University Medical Center Leiden The Netherlands; ^3^ Department of Developmental Biology, Centre of Medical Biotechnology, Faculty of Biology University of Duisburg‐Essen Essen Germany; ^4^ Department of Developmental Biology Washington University School of Medicine St. Louis MO USA; ^5^ Department of Molecular Genetics University of Toronto Toronto Canada; ^6^ Lunenfeld‐Tanenbaum Research Institute, Sinai Health System Toronto Canada

**Keywords:** ADPKD, fibrosis, cell polarity, kidney injury

## Abstract

Autosomal Dominant Polycystic Kidney Disease is characterised by the development of fluid‐filled cysts in the kidneys which lead to end‐stage renal disease (ESRD). In the majority of cases, the disease is caused by a mutation in the *Pkd1* gene. In a previous study, we demonstrated that renal injury can accelerate cyst formation in *Pkd1* knock‐out (KO) mice. In that study, we found that after injury four‐jointed (Fjx1), an upstream regulator of planar cell polarity and the Hippo pathway, was aberrantly expressed in *Pkd1* KO mice compared to WT. Therefore, we hypothesised a role for Fjx1 in injury/repair and cyst formation. We generated single and double deletion mice for *Pkd1* and *Fjx1*, and we induced toxic renal injury using the nephrotoxic compound 1,2‐dichlorovinyl‐cysteine. We confirmed that nephrotoxic injury can accelerate cyst formation in *Pkd1* mutant mice. This caused *Pkd1* KO mice to reach ESRD significantly faster; unexpectedly, double KO mice survived significantly longer. Cyst formation was comparable in both models, but we found significantly less fibrosis and macrophage infiltration in double KO mice. Taken together, these data suggest that *Fjx1* disruption protects the cystic kidneys against kidney failure by reducing inflammation and fibrosis. Moreover, we describe, for the first time, an interesting (yet unidentified) mechanism that partially discriminates cyst growth from fibrogenesis. © 2019 The Authors. *The Journal of Pathology* published by John Wiley & Sons Ltd on behalf of Pathological Society of Great Britain and Ireland.

## Introduction

Autosomal Dominant Polycystic Kidney Disease (ADPKD) is a genetic disease caused in the majority of the cases by a mutation in the *PKD1* gene, which encodes polycystin 1, and in the remaining cases by a mutation in the *PKD2* gene, encoding polycystin 2 1. The hallmark of this disease is the formation of fluid‐filled cysts in the kidneys, which grow slowly and progressively disrupt the renal parenchyma, ultimately leading to kidney failure 1, 2. The exact mechanisms behind cyst formation are still elusive, and effective therapies are still missing, although the Vasopressin V2R antagonist tolvaptan has become recently available for selected patients 3, 4, 5.

Recently, our group showed that a substantial proportion of genes typically deregulated in ADPKD also play roles in injury‐repair mechanisms 6. Indeed, since less than a decade ago, injury has emerged as an important player in cyst formation and progression, and now it is considered a ‘modifier’ of ADPKD 7. Several other groups, and we, have described that both nephrotoxic 8 and ischaemic injury, as well as unilateral nephrectomy 9, 10, 11, 12, were able to speed up cyst formation and progression, reinforcing the link between ADPKD progression and injury. In particular, we identified one gene, *four‐jointed box kinase 1* (*Fjx1*), as an interesting player in these processes. In our study, *Fjx1* showed aberrant expression during both the injury‐repair phase and cyst progression in *Pkd1* KO mice compared with WT mice 8. Moreover, *Fjx1* is implicated with two important pathways normally aberrant in ADPKD: planar cell polarity (PCP) and the Hippo pathway.

Fjx1 is the mammalian homolog of the *Drosophila* protein Fj, discovered for its pivotal role in the correct development of leg joints, wings and eyes 9, 10. Fj regulates the interaction of Fat (Ft) with Dachsous (Ds), which controls PCP signalling, most likely in parallel with the Frizzled signals 10, 11, 12. *Fj* mutant *Drosophila* models have a clear alteration of PCP, whereas *Fjx1* KO mice do not show any morphological defects in the kidneys or other organs 13, 14. However, deleting the target of Fjx1, *Fat4*, leads to loss of PCP in the inner ear, cochlea and the neural tube, and mild cyst formation in mouse kidney. Loss of both, *Fat4* and *Fjx1*, slightly aggravates the phenotype, suggesting that Fjx1 may also act via Fat4‐independent pathways. Yet, the effect of *Fjx1* in a *Pkd1* mutant context is to date unknown 14.

In *Drosophila*, Fj is also an upstream regulator of the Hippo pathway, through its downstream target Ft. The Hippo pathway regulates proliferation and tissue size through the activity of the final effector and transcriptional co‐activator Yorki (Yki) 15, 16. In mammals, there are two *Yki* orthologs: *Yes‐associated protein 1* (*Yap1*) and *transcriptional coactivator with PDZ‐binding motif* (*Wwtr1* or *Taz*). When the Hippo pathway is active, Yap1 and Taz are phosphorylated and retained in the cytoplasm, preventing their nuclear translocation and transcriptional activity. In ADPKD, Yap and Taz activity is upregulated in the cyst lining epithelium as indicated by their nuclear localisation, suggesting a role for this pathway in cyst progression 17. In mammals, regulation of the Hippo pathway by Fat4 has recently been shown in the prenatal heart 18; however, whether this regulatory mechanism also takes place in the kidneys is not clear 14, 19.

This study aimed to investigate the role of Fjx1 during ADPKD progression, particularly after kidney injury and the involvement of the PCP and Hippo pathways. We found that mice that are double KO for *Fjx1* and *Pkd1* display cyst formation comparable to that of single *Pkd1* KO mice but survive longer. This effect was probably not due to differences in PCP and the Hippo pathway, which were unaffected by *Fjx1* deletion, but rather due to reduced fibrosis and macrophage infiltration in the double KO mice. We also found a reduction of fibrosis which was independent of cyst formation. Indeed, in our study, reduced fibrogenesis was caused directly by *Fjx1* deletion and was not an indirect consequence of the improved cystic phenotype.

## Methods

### Animal models

All the animal experiments were evaluated and approved by the local animal experimental committee of the Leiden University Medical Centre and the Commission Biotechnology in Animals of the Dutch Ministry of Agriculture. The kidney‐specific tamoxifen‐inducible *Pkd1*‐deletion mouse model (*Pkd1*‐cKO) and the *Fjx1* ^−/−^ (*Fjx1* KO) has been described previously 13, 20. By cross‐breeding *Pkd1*‐cKO with the *Fjx1* KO mice, we generated the *Fjx1* ^−/−^/*Pkd1*‐cKO double KO mouse model (double KO). Inactivation of the *Pkd1* gene was achieved by oral administration of tamoxifen (Sigma‐Aldrich, Merck KGaA, Darmstadt, Germany) in adult mice (13–14 weeks old). Renal injury was induced a week after gene disruption by a single i.p. injection of S‐(1,2‐dichlorovinyl)‐l‐cysteine (DCVC) or vehicle. Injury was evaluated by measurement of blood urea nitrogen (BUN) level after 40 h, as described 8. More details are provided in supplementary material, Supplementary materials and methods.

### Immunohistochemistry

Formalin‐fixed paraffin‐embedded kidneys were sectioned at 4 μm thickness. Sections were stained with PAS to determine the cystic index (CI) and with picro‐sirius red (PSR) to determine fibrotic index. Kidney sections were also stained for αSMA, F4/80, YAP, pSTAT3, GM130. More details are provided in supplementary material, Supplementary materials and methods.

### RT‐qPCR

Snap‐frozen kidneys were homogenised using Magnalyser technology (Roche, Basel, Switzerland). Total RNA was isolated using Tri‐Reagent (Sigma‐Aldrich). cDNA synthesis was performed using the Transcriptor First Strand cDNA Synthesis Kit (Roche), and qPCR performed using ×2 FastStart SYBR‐Green Master (Roche) according to the manufacturer's protocol. Primer sequences are provided in supplementary material, Table S1. Levels of mRNA were normalised to *Hprt* and fold‐change was used for representation in the graphs.

### Statistical analysis

Data were analysed using ANOVA in GraphPad Prism 8.00 for Windows (GraphPad Software, San Diego, CA, USA) and linear‐mixed effects models in IBM SPSS Statistics for Windows, version 23 (IBM Corp., Armonk, NY, USA).

## Results

### Mice double KO for *Pkd1* and *Fjx1* survive longer after toxic tubular damage compared with mice single KO for *Pkd1*

Inactivation of the *Pkd1* gene was achieved by oral administration of tamoxifen in adult mice. This type of mouse model is characterised by a relatively slow cyst growth that allows having reasonable time windows for the study of the different steps of disease progression.

**Figure 1:**
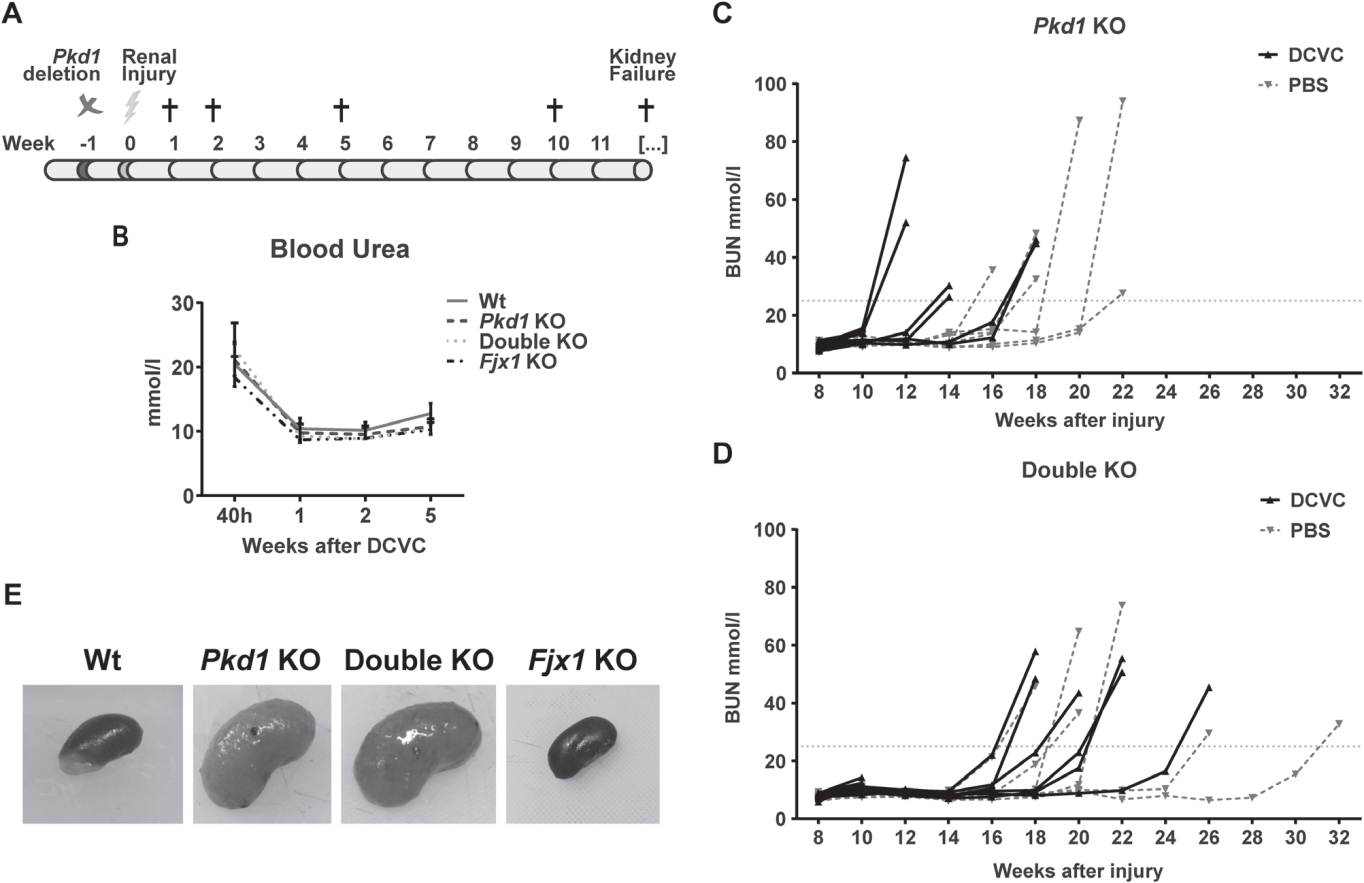
BUN level after DCVC injection and during disease progression. (A) Graphical representation of the mouse experiment pipeline. Adult mice (13–14 weeks old) were fed with 5 mg/day of Tamoxifen for 3 days (week −1). A week later they were injected i.p. with 15 mg/kg of DCVC or PBS as a control (week 0). Mice were sacrificed 1, 2, 5 and 10 weeks after DCVC or PBS injection and at kidney failure, indicated by a rise in BUN over 25 mmol/l. (B) BUN level in the first weeks after DCVC injection. All the genotypes were back to normal level after a week post injury induction. (C) BUN level in *Pkd1* KO mice and (D) in double KO mice with (black solid line) and without (grey dashed line) injury. Each line represents a mouse. Kidney failure was accelerated by DCVC treatment in *Pkd1* KO mice (median DCVC group: 14 weeks; median PBS group: 19 weeks; Mann–Whitney test, *P* value < 0.05) but not in double KO mice (median DCVC group: 20 weeks; median PBS group: 21 weeks), which reached kidney failure significantly later than *Pkd1* KO + DCVC (two‐way ANOVA with Tukey's multiple comparisons test, *P* value < 0.05). (E) Representative whole‐mount kidneys at renal failure (*Pkd1* KO and double KO) or 24 weeks after DCVC (Wt and *Fjx1* KO). *Pkd1* KO and double KO kidneys showed enlarged cystic kidneys, compared to Wt and *Fjx1* KO kidneys which did not show any visible alteration.

We showed previously that upon nephrotoxic injury cyst initiation is faster in mice with *Pkd1* deletion compared with the non‐injured group 8. Using the same injury model, we administered the nephrotoxic compound DCVC to Wt, *Pkd1* KO, *Fjx1* KO and double KO mice (Figure 1A). We used PBS injection as a control (vehicle group). At 40 h after DCVC injections, renal injury was confirmed by a substantial rise in the BUN level in all mice, which returned to baseline after 1 week, suggesting a full recovery of the kidney function with no differences among the genotypes (Figure 1B).

*Pkd1* KO mice injected with DCVC reached end‐stage renal disease (ESRD) around 14 weeks after injury. This was significantly earlier than in the vehicle group, which survived for about 19 weeks, in accordance with previously generated data (Figure 1C) 8. Surprisingly, we observed that the double KO mice did not show a difference between DCVC and vehicle treatment with a median survival of 20 and 21 weeks, respectively (Figure 1D). When compared with *Pkd1* KO mice, double KO mice survived significantly longer after injury, indicating that the lack of *Fjx1* improved survival of double KO mice upon renal damage.

Both Wt and *Fjx1* KO mice subjected to renal injury did not develop cysts still 24 weeks after DCVC, the time point when mice were sacrificed (Figure 1E and data not shown).

### Knocking‐out *Fjx1* in *Pkd1* mutant mice does not affect cyst formation

**Figure 2:**
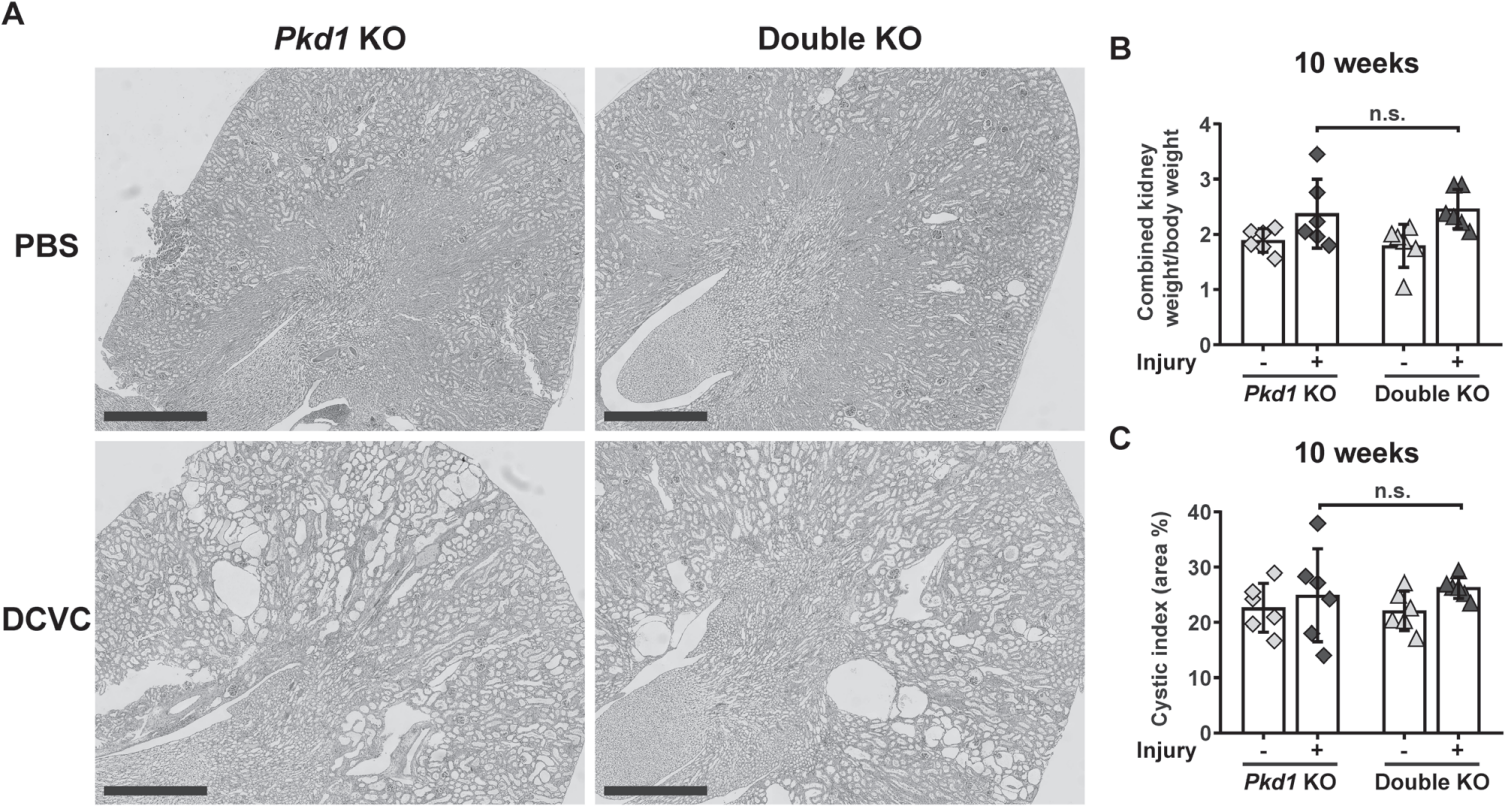
Cysts formation at 10 weeks after DCVC. (A) Representative PAS staining of *Pkd1* KO and double KO mice kidneys at 10 weeks after DCVC, showing comparable cyst formation in the two genotypes. Scale bars, 1 mm. (B) Evaluation of kidney size at the 10 week time point in *Pkd1* KO and double KO mice with and without injury using two kidney weight/body weight ratio. (C) Cystic index at the 10 week time point in *Pkd1* KO and double KO mice with and without injury. Each symbol shows data from one mouse. Mean ± SD. Two‐way ANOVA with Tukey's multiple comparisons test.

**Figure 3:**
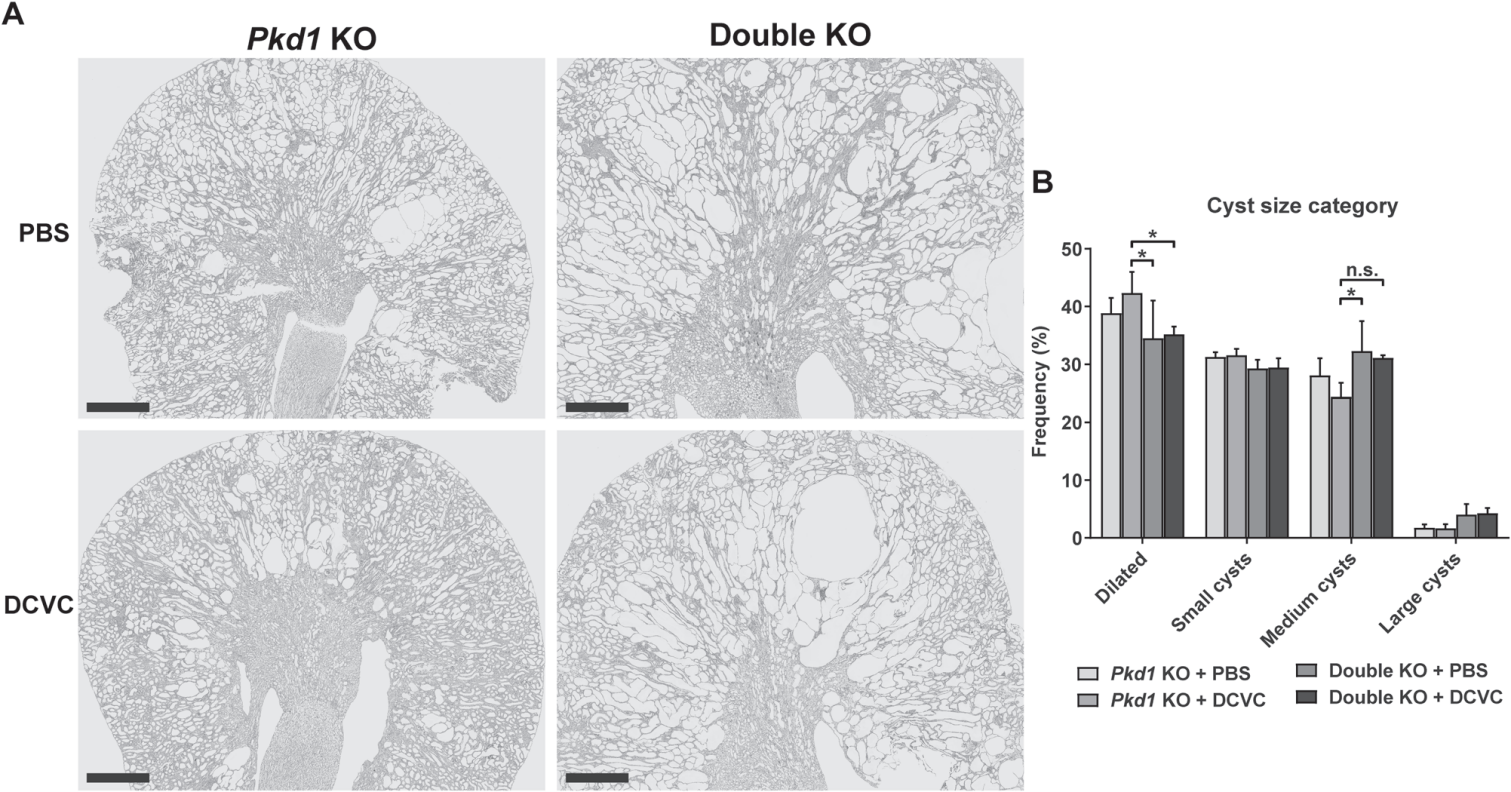
Cysts size at kidney failure. (A) Representative PAS staining of kidneys of *Pkd1* KO and double KO mice at kidney failure. Scale bars, 1 mm. (B) Quantification of cysts size frequency in *Pkd1* KO and double KO mice with and without injury. Data represent the mean of 4 mice ± SD. Three‐way ANOVA (*P* value < 0.0001) with Tukey's multiple comparisons test. *P value < 0.05.

Since renal injury accelerates cyst formation in *Pkd1* KO mice 8, 21, 22, 23, 24, we wondered whether prolonged survival observed in the double KO group treated with DCVC could be due to delayed cyst initiation. We measured the cystic index in kidneys from *Pkd1* KO and double KO mice at 10 weeks after DCVC injection when mice start to show a mild cystic phenotype. We compared *Pkd1* KO and double KO mice with and without DCVC, and did not find any difference in the cystic index, and two kidneys weight to body weight (2KW/BW) ratios between the genotypes at this time point (Figure 2A–C). Thus, the initiation of cyst formation is not different in the two models, suggesting a role in cyst growth. This was also evident when ESRD kidneys were compared. Indeed, *Pkd1* KO mice injected with DCVC, which reached ESRD faster, had a shorter phase of cyst growth and displayed mainly small cysts at kidney failure. Conversely, double KO mice, which had a slower progression to ESRD and therefore a longer phase of cyst growth, showed frequently larger cysts (Figure 3A,B). Thus, our data suggest that Fjx1 is not directly involved in cyst formation and that prolonged survival of the double KO mice cannot be explained by delayed cyst formation.

### Chronic injury caused by cyst formation leads to differences in injury markers expression, fibrosis and inflammatory responses in double KO compared with *Pkd1* KO mice

Cyst formation is accompanied by inflammation and fibrosis which ultimately lead to complete loss of renal function. As changes in these processes might affect survival, we analysed whether *Fjx1* deletion altered the expression of injury markers, fibrosis and inflammation.

**Figure 4:**
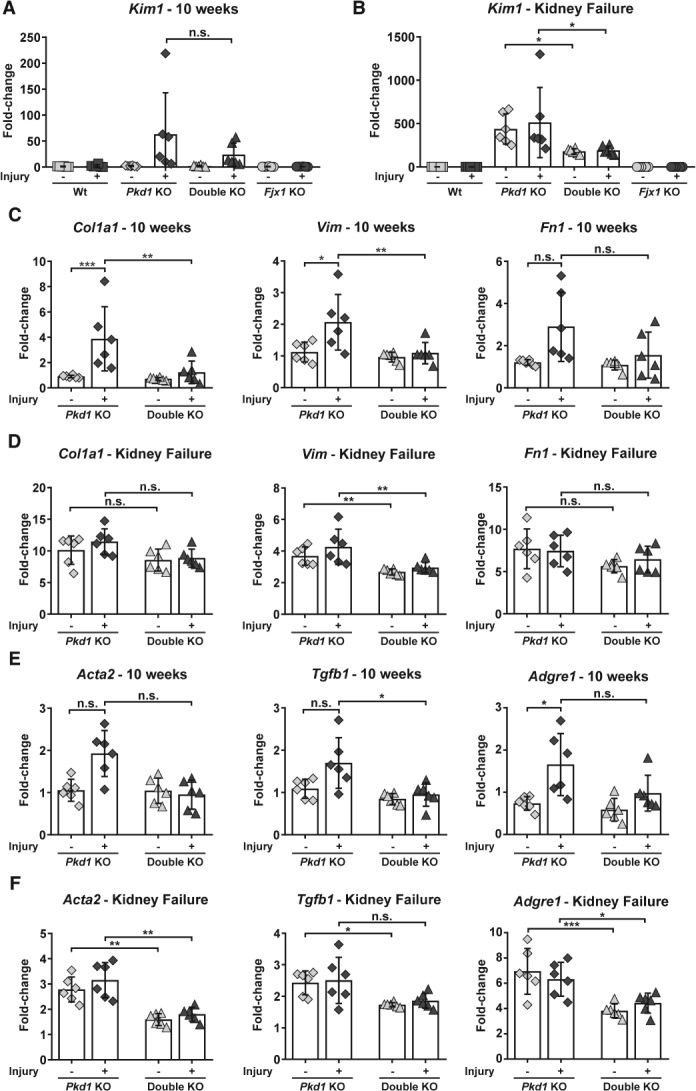
Expression of genes involved in injury‐repair in *Pkd1* KO and double KO mice. (A) Gene expression of *Kim1* (*Havcr1*) at 10 weeks after DCVC injection. Both *Pkd1* KO and double KO have a significant increase of *Kim1* expression compared to the PBS groups and the Wt and *Fjx1* KO with and without injury (significance not shown on graph), but not compared to each other. (B) Gene expression of *Kim1* at kidney failure after DCVC injection (significance to Wt and *Fjx1* KO not shown on graph). (C) *Col1a1*, *Vim* and *Fn1* mRNA levels at 10 weeks after DCVC injection. (D) *Col1a1*, *Vim* and *Fn1* mRNA levels at kidney failure. (E) *Acta2*, *Tgfb1* and *Adgre1* (F4/80) mRNA levels at 10 weeks after DCVC injection and (F) at kidney failure. Each symbol shows data from one mouse. Mean ± SD. Two‐way ANOVA with Tukey's multiple comparisons test. *P value < 0.05; **P value < 0.01; ***P value < 0.001.

The expression of the well‐established kidney injury molecule *Kim1* (*Havcr1*) 25 was analysed using RT‐qPCR. We observed increased *Kim1* expression already at 10 weeks after DCVC injection in *Pkd1* KO and in double KO mice, a time‐point when dilation of tubules and small cysts was evident. In contrast, Wt and *Fjx1* KO mice, which did not develop a renal phenotype after DCVC injection, did not show increased *Kim1* expression, reinforcing the idea of cyst‐induced chronic injury (Figure 4A). Interestingly, at kidney failure, *Kim1* expression was significantly higher in *Pkd1* KO mice compared to double KO (Figure 4B).

**Figure 5:**
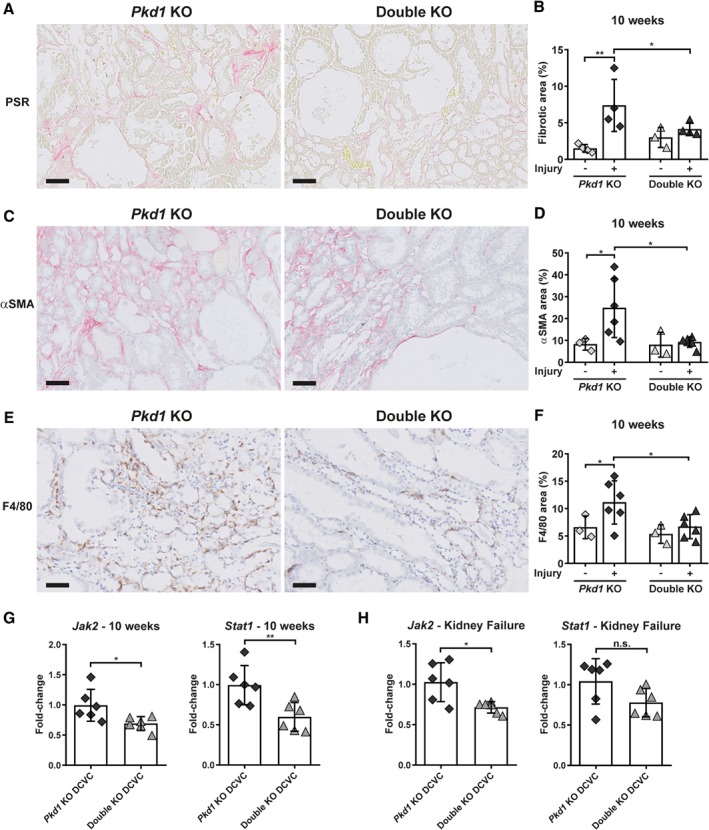
Fibrosis, fibroblast activation and macrophage infiltrate in Pkd1 KO and double KO mice. (A) Representative PSR staining of *Pkd1* KO and double KO mouse kidney at 10 weeks after DCVC. Scale bars, 50 μm. (B) Quantification of PSR staining in the cortico‐medullary region. (C) Representative αSMA staining of *Pkd1* KO and double KO mice at 10 weeks after DCVC. Scale bars, 50 μm. (D) Quantification of αSMA staining in the cortico‐medullary region. (E) Representative F4/80 staining of *Pkd1* KO and double KO mice at 10 weeks after DCVC. Scale bars, 50 μm. (F) Quantification of F4/80 staining in the cortico‐medullary region. Each symbol is a mouse and data represent the mean ± SD. Two‐way ANOVA with Tukey's multiple comparisons test. *P value < 0.05; **P value < 0.01. (G) *Stat1* and *Jak2* mRNA levels at 10 weeks after DCVC in *Pkd1* KO and double KO mice. (H) *Stat1* and *Jak2* mRNA levels at kidney failure in *Pkd1* KO and double KO mice treated with DCVC. Each symbol shows data from one mouse. Mean ± SD. Unpaired *t*‐test. *P value < 0.05; **P value < 0.01.

When we analysed fibrogenesis at 10 weeks after DCVC and kidney failure, expression of alpha‐1 type I collagen (*Col1a1*) and Vimentin (*Vim*) was significantly reduced in the double KO compared to *Pkd1* KO at 10 weeks after DCVC, both at the mRNA (Figure 4C,D) and protein levels (Figure 5A,B); expression of Fibronectin (*Fn1*) showed a similar trend (Figure 4C,D). Interestingly, in *Pkd1* KO mice the expression of these genes was significantly correlated with kidney size, but this was not observed in the double KO mice, suggesting that in these mice cyst progression and fibrosis are two independent events (see supplementary material, Figure S1). Likewise, the expression of transforming growth factor beta‐1 (*Tgfb1*) was significantly less in double KO compared to *Pkd1* KO mice at 10 weeks and showed a similar trend at kidney failure. Also, double KO mice at 10 weeks exhibited a trend for lower alpha‐smooth muscle actin (*Acta2*) transcript levels and significantly less αSMA‐positive area (Figures 4E,F and 5C,D).

To characterise the inflammatory response, we looked at the level of transcripts for the macrophage marker *Adgre1* (F4/80) and found this to be significantly less abundant in double KO compared to *Pkd1* KO mice at kidney failure (Figure 4E,F). At 10 weeks after DCVC *Adgre1* showed a trend but at the protein level F4/80 expression was significantly reduced in double KO mice (Figure 5E,F). We also assessed the expression of *Jak2* and *Stat1*, involved in the transduction of a series of signals, such as growth factors and cytokines, in response to injury 26. We found significantly lower expression in double KO compared to *Pkd1* KO mice (Figure 5G,H). On the other hand, Stat3 activation, known to be involved in cyst growth 27, was not significantly different between the two genotypes, supporting the idea that Fjx1 role is related to the inflammatory/fibrotic response and not to cyst formation (see supplementary material, Figure S2). These results indicate that the lack of *Fjx1* leads to a reduced inflammatory/fibrotic response which translates into a longer survival after DCVC administration.

### Investigation of pathways involved in renal fibrosis

**Figure 6:**
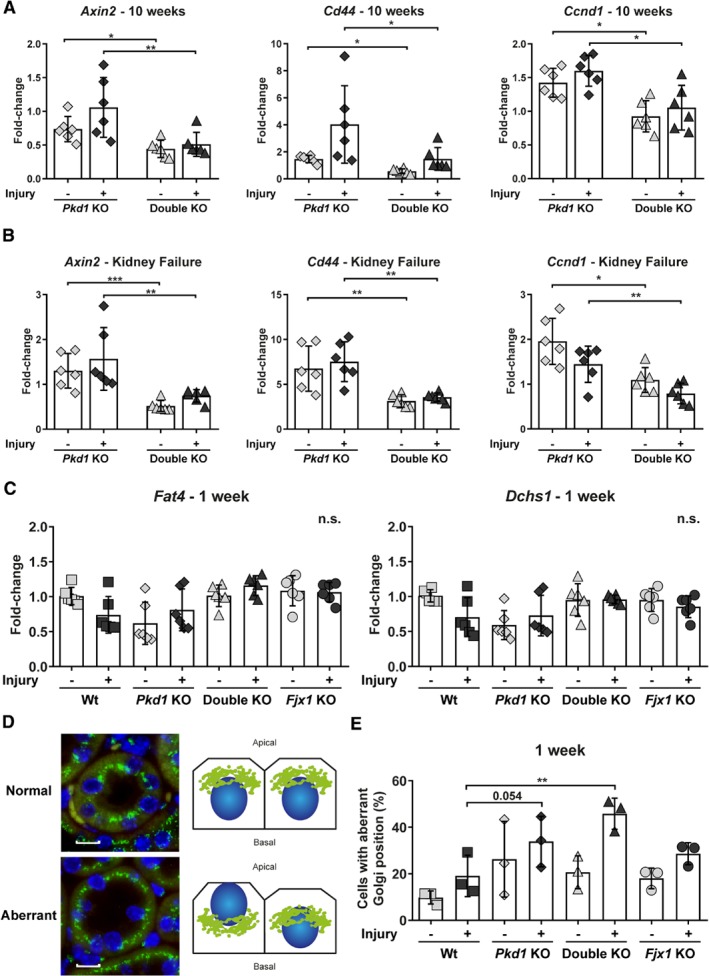
Expression of Wnt pathway target genes and expression level of Fjx1 targets and Golgi position in tubular cells. (A) *Axin2*, *Cd44* and *Ccnd1* mRNA levels at 10 weeks after DCVC injection. (B) *Axin2*, *Cd44* and *Ccnd1* mRNA levels at kidney failure. (C) *Fat4* and *Dchs1* mRNA levels at 1 week after DCVC injection. Each symbol shows data from one mouse. Mean ± SD. Two‐way ANOVA with Tukey's multiple comparisons test. *P value < 0.05; **P value < 0.01; ***P value < 0.001. (D) Representative GM130 (green) staining on kidney tissue. Nuclei are stained in blue. In the normal situation, the Golgi body is positioned in a peri‐centrosomal position at the top of the nucleus towards the lumen of the tubules, but after injury we often observed altered Golgi position. Scale bars, 10 μm. (E) Golgi position has been scored from 1 (normal position) to 3 (very abnormal position) in the round shaped tubules in the cortico‐medullary region. Results are represented as the percentage of aberrant Golgi position (score ≥ 2.5) per tubules. Each symbol is the mean ± SD of about 90 tubules scored in a mouse. Two‐way ANOVA with Fisher's LSD. **P value < 0.01.

We also studied the expression of key genes of several pathways known to be involved in renal fibrosis, such as Notch 28, 29, Hedgehog 30, 31, Wnt 32, 33, hypoxia 34, 35, 36, 37, 38 and Egf 39, 40, 41. However, we could not find any differences between the double KO and *Pkd1* KO mice, except for target genes of Pdgfb‐ (see supplementary material, Figure S3) and Wnt. Indeed, *Axin2*, *Cd44*, *Ccnd1* (Figure 6A,B) and to certain extent *Myc* (see supplementary material, Figure S4), showed significantly lower expression in double KO compared with *Pkd1* KO mice, both at 10 weeks after DCVC and at kidney failure, suggesting a reduced activation of the canonical Wnt signalling in the absence of *Fjx1*.

### Mice double KO for *Pkd1* and *Fjx1* show less sensitivity to DCVC‐induced injury than mice single KO for *Pkd1*

To further investigate the role of Fjx1 in injury, we performed a pilot experiment in which mice treated with DCVC were sacrificed after 24, 48 and 72 h, i.e. during the nephrotoxin‐induced acute injury phase. At all time‐points, we found a trend consistent with that observed in the cyst‐induced chronic injury, showing that *Kim1* expression was less in double KO mice than in *Pkd1* KO. At 1 week after DCVC *Kim1* expression was strongly reduced in both genotypes suggesting that the DCVC‐induced acute injury is largely repaired in the first week (see supplementary material, Figure S5). In line with the findings from the chronic injury experiments, the expression of genes involved in fibrogenesis, such as *Cola1a*, *Vim* and *Fn1*, was lower in double KO compared with *Pkd1* KO mice (see supplementary material, Figure S5).

Together with the results observed during the cyst‐induced chronic injury, these data suggest that the lack of *Fjx1* leads to a reduced sensitivity to DCVC‐induced injury.

### PCP is altered in Pkd1 mutant mice after injury but is not significantly affected by the lack of *Fjx1*

Tissue injury causes inversion or loss of PCP in epithelial cells, and this recovers during the repair phase 42. *Fj* has been described as an important PCP gene in *Drosophila* as is *Fjx1* in mammals, in particular in the regulation of the brain architecture 13 and inner ear polarity 14. Therefore we decided to characterise renal PCP at 1 week after injury in *Pkd1* KO and double KO mice.

The levels of expression of *Fat4* and its ligand *Dchs1* 43 were unchanged by *Fjx1* KO (Figure 6C) suggesting that, at the expression level, the Ft/Ds PCP pathway was unaltered in kidneys of mutant mice. We also used the position of the Golgi body to assess the degree of polarity perturbation in tubular cells (Figure 6D). Although Golgi position is not a direct read‐out of PCP core proteins, it is found to be aberrant when PCP genes are knocked‐out 44, and is also associated with loss of directed secretion, cell polarity and wound healing capacity 45.

We confirmed that altered polarity was associated with loss of *Pkd1* and kidney injury already at the pre‐cystic stage, with a significantly more aberrant Golgi position in *Pkd1* KO and double KO mice compared with Wt and *Fjx1* KO. However, we could not identify any difference between double KO and *Pkd1* KO or between Wt and *Fjx1* KO (Figure 6E), indicating that *Fjx1* did not contribute to an altered PCP.

### The effect of *Fjx1* on injury response is not mediated by the Hippo pathway

Fjx1 is thought to be an upstream regulator of the Hippo pathway through the activity of Fat4 46. The Hippo pathway is pivotal in the regulation of organ growth, tissue renewal and regeneration 47 but is also deregulated in ADPKD 17. Therefore, we investigated this pathway in *Pkd1* KO and double KO mice after DCVC treatment.

Immunostaining of kidney sections for Yap confirmed the pattern described previously in our lab, with increased nuclear localisation of Yap in the cystic epithelium 17. However, we could not detect any significant difference between double KO and *Pkd1* KO. Also, neither mRNA levels of *Yap1* and its paralog *Taz*, nor their transcriptional targets, *Amotl2*, *Cyr61*, *Wtip*, *Ctgf*, *Ajuba* (see supplementary material, Figure S6), showed any significant difference among genotypes. This suggests that despite the clear nuclear localisation of Yap in the cystic epithelium this pathway is not responsible for the difference in survival between *Pkd1* KO and double KO mice.

Taken together, these data indicate that knocking‐out *Fjx1* does not affect PCP and the Hippo pathway in the kidneys. Therefore, the differences observed in response to injury in double KO mice cannot be explained by the effect of *Fjx1* on one of its canonical targets but suggests the existence of other, yet unknown, Fjx1 targets.

## Discussion

In this study we showed that nephrotoxic injury can accelerate disease progression in *Pkd1* KO mice but that this effect is abolished in the absence of *Fjx1* expression, allowing the *Pkd1*/*Fjx1* double KO mice to survive on average 5 weeks longer than the single *Pkd1* KO mice. Interestingly, the initiation of cyst formation and cyst growth were not different among the two models, as shown by 2KW/BW ratios and the cystic index. At 10 weeks after DCVC and kidney failure, however, we observed a reduction in injury marker expression together with reduced fibrosis and macrophage infiltration in *Pkd1* KO mice compared with double KO. Therefore, these data suggest that Fjx1 does not play a critical role in cyst formation and expansion but seems to be involved in the fibrotic and inflammatory response to injury. As a result, the mice lacking both, *Fjx1* and *Pkd1*, have less fibrosis, which leads to a slower progression to ESRD and longer survival.

Fj is – together with the Ft‐Ds cassette – part of a signalling complex that is involved in the regulation of PCP in *Drosophila* 48, 49, 50, 51. Nevertheless, the absence of *Fjx1* did not alter PCP in the kidneys when compared with Wt mice, or in double KO compared to *Pkd1* KO mice. Although we observed significant deregulation of PCP in pre‐cystic kidneys after injury in both single *Pkd1* mutant mice and double KO, the additional deletion of *Fjx1* did not further change the PCP phenotype in the *Pkd1* KO. These results are consistent with the published work of Probst *et al* 13 showing that *Fjx1* KO mice do not have aberrant PCP in the kidneys but show only defects in neuronal branching. An effect on renal PCP was only seen after knocking‐out *Fat4*, a target of Fjx1, suggesting a more indirect effect of Fjx1 on PCP in mammals 14, 19. We showed in a previous study that PCP is impaired in *Pkd1* KO mice but not in Wt mice after injury, and that *Pkd1* KO mice injected with DCVC also develop cysts earlier when compared with the PBS group 8. Whether pre‐cystic alterations of PCP are critical for cyst formation is still controversial. Several studies are suggesting that PCP and cilium‐associated control of oriented cell division (OCD) as well as convergent extension (CE) are necessary during renal tubular morphogenesis and also during proliferation phases in adult kidneys. Alterations of both OCD and CE are involved in PKD 23, 52, 53, 54. However, there are also studies showing that alterations in OCD and CE occur only after cyst formation, or that mutations of PCP‐core proteins do not result in cyst formation 55, 56. This means that simple alteration of PCP is not sufficient to start cyst formation but disrupted PCP together with other events, such as injury, presumably increases the likelihood of cyst initiation.

Another pathway altered in ADPKD is the Hippo pathway, which is also regulated via the Fjx1 target Fat4. In particular, the pathway's effectors Yap1 and Taz have been associated with cyst formation. We showed in the past that Yap1 accumulates in the nuclei of the cyst‐lining epithelium 8, 17, and other groups showed how deregulations of Yap1 activity could induce cyst formation in Zebrafish models 57, 58. Moreover, knocking‐out *Taz* in mice leads to glomerular and proximal tubular cyst formation 59, 60, 61. Nevertheless, we did not see an effect of Fjx1 on the Hippo pathway when comparing *Pkd1* KO and double KO mice. *Yap1* and *Taz* levels, as well as the levels of several of their target genes, were comparable in the two genotypes throughout disease progression. Also, *Fat4* levels were unchanged by *Fjx1* deletion (data not shown). Currently, clear proof that the Fjx1‐Fat4‐Dchs cassette interaction controls PCP and Hippo pathway in kidneys is still missing 14, 19, 62.

Once cysts start to form and expand, they compress the surrounding tissue, compromise the normal tubular structure and also interfere with the extracellular compartment. This is accompanied by the expression of injury markers and activation of transcription factors like Stat3, Creb and ERK, known to be involved in ADPKD pathogenesis, and with an increased likelihood of more cyst formation 22, 27, 63, 64. All these cues are perceived by the organ like a constant injury insult and accompanied by a fibrotic and inflammatory response. Concomitantly, a severe cystic phenotype is associated with renal function decline due to the accumulation of fibrosis and inflammatory infiltrates, which interfere with normal organ function 65, 66. Nonetheless, it is unclear whether inflammation and fibrosis are responsible for or just a consequence of cyst formation. In our study, we observed separation between cyst formation and fibrotic response when *Fjx1* is inactivated. Indeed double KO mice had significantly reduced fibrosis and leukocytes infiltrates compared with *Pkd1* KO mice even though cyst formation was comparable. We could exclude the involvement of some fibrosis‐related pathways, such as Notch, Hedgehog, hypoxia and Egfr signalling, while we found a significant reduction of expression of *Pdgfb*, *Tgfb1*, *Jak2* and *Stat1*, and Wnt pathway target genes in double KO compared to *Pkd1* KO mice. Considering the well described role of Tgfb and Wnt pathways in renal fibrosis 32, 33, 67, 68, it is plausible to think that they might be responsible for the reduced fibrosis observed in double KO mice. Indeed, Tgfb can regulate the expression of *Pdgfb* 69, *Fn1* and type I collagen 70, 71 all found downregulated in double KO mice. Similarly, Wnt targets *Axin2* 72, *Cd44* 73, *Ccnd1* 74, 75 and *Myc* 76 were lower in double KO mice. Further studies are required to link Fjx1 with the Tgfb and Wnt pathways mechanistically. An interesting connection between Fjx1 and Jak/Stat pathways has been described in the literature, with Fj as the effector of the pleiotropic pathway Jak/Stat in *Drosophila* 10. Although it is tempting to speculate that this might be the route through which Fjx1 modulates the injury response, it is more likely that reduced *Jak2*/*Stat1* levels in the double KO mice mirror a reduced inflammatory response. Overall, these data suggest that Fjx1 is involved in the fibrotic/inflammatory response after injury. We also showed that a different response to injury in the double KO mice could also play a role during the acute injury phase between 24 and 72 h after DCVC injection. This is not surprising, considering that *Fjx1* is mainly expressed in the developing kidneys while its expression is almost absent in adult kidneys 77. Indeed, as for many other developmental genes, injury causes an increase in expression of *Fjx1* 8. Yet, the mechanism through which Fjx1 is influencing these processes is still unclear.

As the function of Fjx1 is still obscure, we cannot exclude that, besides the canonical targets Ft and Ds, additional direct targets of Fjx1 exist. This is because Fjx1 is a Golgi secretory pathway kinase and therefore likely involved in many biological processes, as already shown for its closely related homolog Fam20C 78, 79. Additionally, Fjx1 protein undergoes partial proteolytic cleavage at the N‐terminus, with the secretion of the resultant fragment that can function as signalling ligand, influencing surrounding cells. Fjx1 fusion protein experiments have shown several Fjx1 binding sites present in different organs, including kidneys 77. Therefore, a better understanding of Fjx1 functions in mammals might help to explain the effect we unveiled on fibrogenesis.

In conclusion, we show that cyst progression and fibrosis in *Pkd1*/*Fjx1* double KO mice are partially uncoupled and demonstrate a new, yet undefined, role of Fjx1 in fibrosis, ultimately resulting in longer survival. Unveiling the underlying molecular mechanism might open the path for future therapies that can specifically target injury‐induced fibrosis, and could not only help to slow down ADPKD, but also the progression of other chronic kidney diseases.

## Author contributions statement

CF contributed to the concept design, data acquisition, data interpretation and writing paper. HH contributed to the concept design. DJMP contributed to the concept design, data interpretation and writing paper. KAMV contributed to the histopathology. MS contributed to the data interpretation and manuscript reviewing. AV and HMcN contributed to the mice, data discussion and manuscript reviewing.

**SUPPLEMENTARY MATERIAL ONLINE**

**Supplementary materials and methods**

**Figure S1.** Correlation of fibrosis and kidney size in *Pkd1* KO and *Pkd1*/*Fjx1* double KO mice

**Figure S2.** Expression of pSTAT3 in Pkd1 KO and double KO mice

**Figure S3.** Investigation of pathways involved in renal fibrosis at 10 weeks after injection of the nephrotoxic compound DCVC

**Figure S4.** Wnt pathway target Myc

**Figure S5.** Injury and fibrotic genes expression at early time points after injury

**Figure S6.** Hippo Pathway activation in Pkd1 KO and Pkd1/Fjx1 double KO mice

**Table S1.** List of mouse qPCR primer sets used

## Supporting information


**Supplementary materials and methods**

**Figure S1.** Correlation of fibrosis and kidney size in *Pkd1* KO and *Pkd1*/*Fjx1* double KO mice
**Figure S2.** Expression of pSTAT3 in Pkd1 KO and double KO mice
**Figure S3.** Investigation of pathways involved in renal fibrosis at 10 weeks after injection of the nephrotoxic compound DCVC
**Figure S4.** Wnt pathway target Myc
**Figure S5.** Injury and fibrotic genes expression at early time points after injury
**Figure S6.** Hippo Pathway activation in Pkd1 KO and Pkd1/Fjx1 double KO mice

**Table S1.** List of mouse qPCR primer sets used
