# Supplementary material for: Four‐jointed knock‐out delays renal failure in an ADPKD model with kidney injury
Source: J Pathol. 2019 Jun 17;249(1):114–25. doi: 10.1002/path.5286 (PMC6772084; doi:10.1002/path.5286)
Supplement: Supplementary file 1 — Supplementary materials and methods [file PATH-249-114-s003.docx]

**Four-jointed knock-out delays renal failure in an ADPKD model with kidney injury**

Formica C *et al*. *J Pathol* DOI: 10.1002/path.5286

**Supplementary materials and methods**

Reference numbers refer to the main text list

**Animal Models**

The kidney specific tamoxifen-inducible *Pkd1*-deletion mouse model (*Pkd1*-cKO) has been described previously [20]. The *Fjx1*^-/-^ (*Fjx1* KO) were generated via insertion of the *LacZ* gene followed by a PGK-neo resistance cassette in the single exon of *Fjx1* gene, as described previously [13]. This results in a germline disruption of *Fjx1*. By cross-breeding the kidney specific tamoxifen-inducible *Pkd1*-deletion mice (*Pkd1* KO) with the *Fjx1* KO mice, we generated the *Fjx1*^-/-^/*Pkd1*-cKO double KO mouse model (double KO). Control mice (Wt) carry the *Lox*P site that flanks *Pkd1* exons 2-11 but miss the tamoxifen-inducible Cre recombinase (*Pkd1*^lox,lox^). Inactivation of the *Pkd1* gene was achieved by oral administration of tamoxifen (5 mg/day, 3 consecutive days) in adult mice that were between 13 to 14 weeks old. Only male mice were used for all the experimental groups. The *Fjx1* KO and the Wt mice also received tamoxifen (Sigma-Aldrich, Merck KGaA, Darmstadt, Germany). Renal injury was induced a week after gene disruption by a single intraperitoneal (i.p.) injection of S-(1,2-dichlorovinyl)-L-cysteine (DCVC) (15 mg/kg) or vehicle. Injury was evaluated by measurement of blood urea level after 40 hours, as described previously [8].

Mice were sacrificed at 24, 48 and 72 h after DCVC injection to study acute injury; at 1, 2, 5 and 10 weeks after DCVC injection to study injury/repair and disease progression and when reaching renal failure indicated by urea levels in the blood equal or over 25 mmol/l. Blood urea nitrogen (BUN) level assessment has been described previously [8]. At sacrifice, mice were weighed; then kidneys were collected and weighed to calculate the 2 kidneys weight to body weight ratios (2KW/BW).

**IHC, Golgi position, Cystic and Fibrotic indices**

Formalin-fixed paraffin-embedded kidneys were sectioned at 4 µm thickness. Section stained with Periodic acid-Schiff (PAS) staining were used to determine cystic index (CI). CI is measured as the ratio of cystic area over the total parenchyma area using ImageJ software (open source software; National Institutes of Health, Bethesda, MD, USA) and expressed as a percentage. Sections stained with Picro-Sirius Red (PSR) staining were used to determine fibrotic index (FI). FI was calculated using a designed colour palettes and Photoshop software (Adobe Systems, Inc., San Jose, CA, USA). First a palette was used to remove the pixels of the renal outline area and of the cystic and tubular areas. Then a second palette was used to remove the pixels of the kidney parenchyma except those coloured by the PSR staining. Big arteries were excluded manually. The ratio of PSR positive pixels over the total parenchyma pixels was expressed as a percentage and indicated as FI. The same analysis was used to calculate the area positive for alpha Smooth Muscle Actin (αSMA) and for the area positive for F4/80. These antibodies were used for the IHC: rabbit anti αSMA-AP (1:50; Sigma-Aldrich #A5691); rat anti-F4/80 (1:250; AbD Serotec now Bio-Rad Antibodies, Hercules, CA, USA # MCA497GA); rabbit anti-Yap (1:800; Cell Signaling Technology, Danvers, MA, USA #14074), rabbit anti-pStat3 (1:75; Cell Signaling Technology #9145); mouse anti-GM130 (1:500; BD Bioscience, Franklin Lakes, NJ, USA #610822). Anti-rabbit envision HRP kit (Dako, Agilent, Santa Clara, CA, USA) or anti-rat Immpress HRP kit (Vector Laboratories, Burlingame, CA, USA) or Alexa 488 goat anti-mouse IgG1 (1:200; Invitrogen, Thermo Fisher Scientific, Waltham, MA, USA #A-21121) were used as secondary antibodies.

The frequency of cyst size was calculated using ImageJ software. Images of whole kidneys stained with PAS staining were used. The area of the renal outline was removed and then the area of the tubules lumen was measured in pixels and divided in four groups: Normal tubule or mildly dilated tubules up to 1000 pixels; small cysts between 1000 and 2000 pixels; medium cysts between 2000 and 10000 pixels; big cysts more than 10000 pixels.

For the evaluation of the Golgi position as a read-out of the PCP we used a mouse anti-GM130 followed by a secondary Alexa 488 Goat Anti-mouse IgG1 and mounted with Vectashield with DAPI (Vector Laboratories) to visualise the nuclei. From each individual kidney we selected at least 90 tubules with a circularity ≥ 0.995, which was evaluated by ImageJ software. We scored the Golgi position in relation to the nucleus from 1 to 3, with 1 being the normal peri-centrosomal position at the top of the nucleus towards the lumen, and 3 being very aberrant at the bottom of the nucleus; a score ≥ 2,5 was considered aberrant and the total count was normalised to the number of cells per tubule and expressed as a percentage.
