# Supplementary material for: Four‐jointed knock‐out delays renal failure in an ADPKD model with kidney injury
Source: J Pathol. 2019 Jun 17;249(1):114–25. doi: 10.1002/path.5286 (PMC6772084; doi:10.1002/path.5286)
Supplement: Supplementary file 2 — Figure S1. Correlation of fibrosis and kidney size in Pkd1 KO and Pkd1/Fjx1 double KO mice Figure S2. Expression of pSTAT3 in Pkd1 KO and double KO mice Figure S3. Investigation of pathways involved in renal fibrosis at 10 weeks after injection of the nephrotoxic compound DCVC Figure S4. Wnt pathway target Myc Figure S5. Injury and fibrotic genes expression at early time points after injury Figure S6. Hippo Pathway activation in Pkd1 KO and Pkd1/Fjx1 double KO mice [file PATH-249-114-s001.pdf]

Four-jointed knock-out delays renal failure in an ADPKD model with kidney injury

Formica *et al.* J Pathol DOI: 10.1002/path.5286

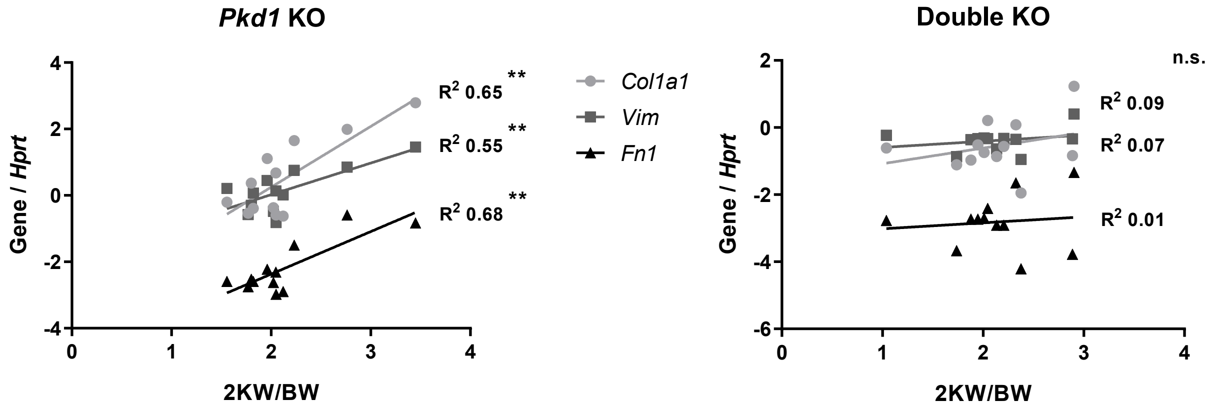

Figure S1. Correlation of fibrosis and kidney size in *Pkd1* KO and *Pkd1*/*Fjx1* double KO mice.
Linear regression of 2KW/BW ratio and gene expression normalized on *Hprt* of *Col1a1*, *Vim* and *Fn1* at 10 weeks time point. ** P value <0.01

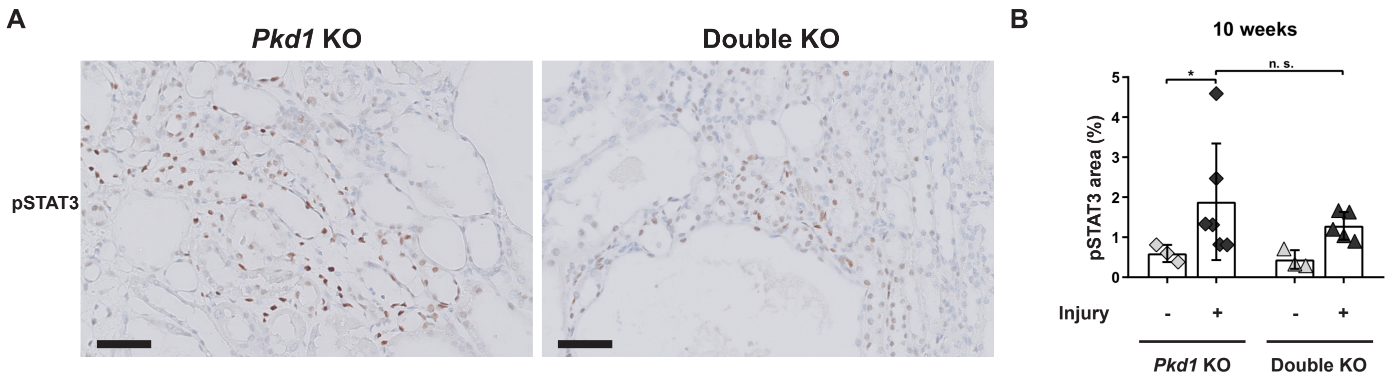

Figure S2. Expression of pSTAT3 in *Pkd1* KO and double KO mice. (A) Representative immunostaining for pSTAT3 at 10 weeks after injection of the nephrotoxic compound S-(1,2-dichlorovinyl)-L-cysteine (DCVC) in *Pkd1* KO and *Pkd1*/*Fjx1* double KO mice kidneys, indicated as ± injury. Scale bars, 50 µm. (B) Quantification of pSTAT3 staining. Each symbol shows data from one mouse. Mean ± SD. Two-way ANOVA with Tukey's multiple comparisons test. * P value <0.05.

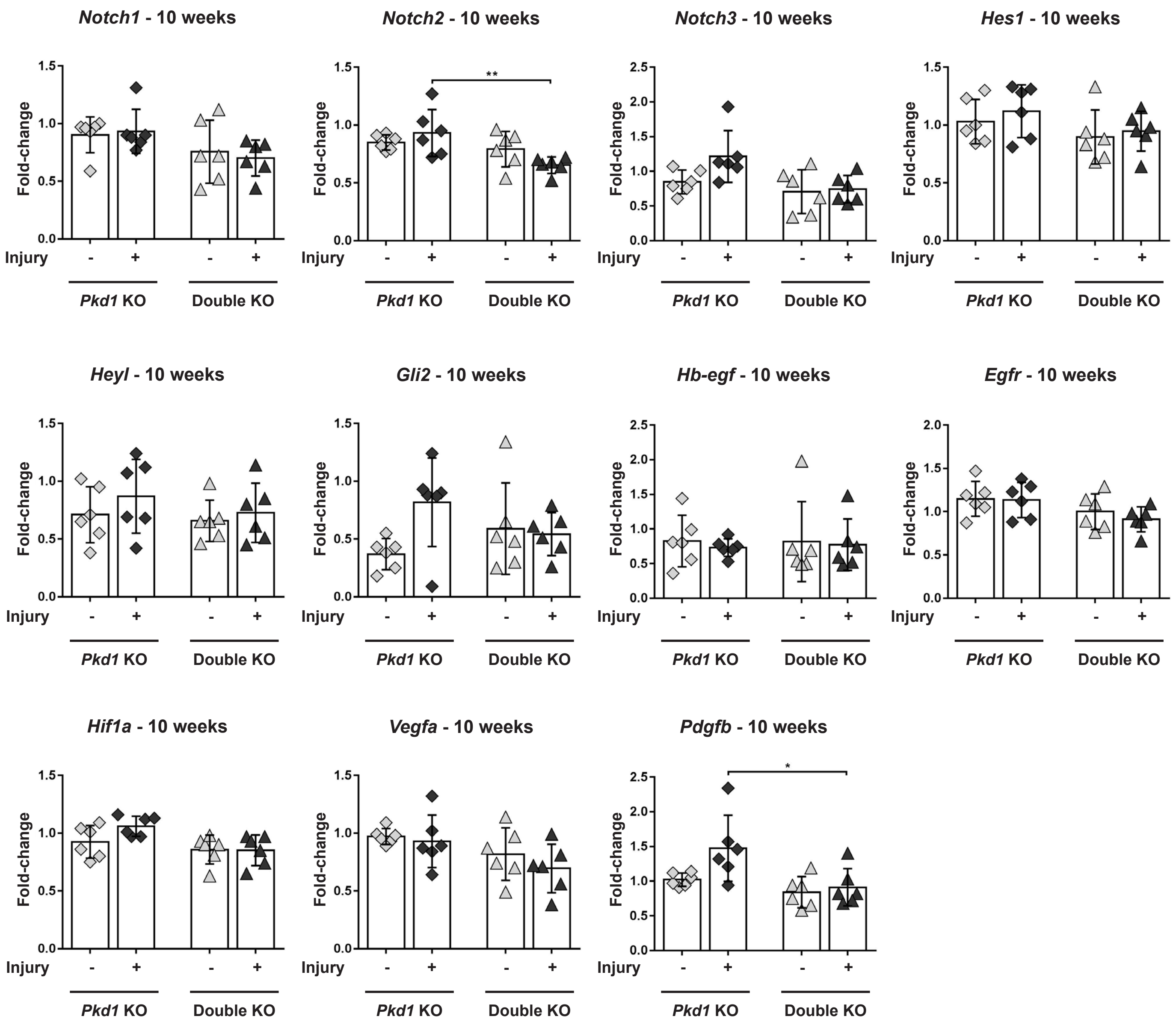

Figure S3. Investigation of pathways involved in renal fibrosis at 10 weeks after injection of the nephrotoxic compound S-(1,2-dichlorovinyl)-L-cysteine (DCVC). Gene expression in *Pkd1* KO mice and *Pkd1*/*Fjx1* double KO mice with or without renal injury induced via injection of DCVC, of Notch target genes (*Notch1*, *Notch2*, *Notch3*); Hedgehog target genes (*Hes1*, *Hey1*, *Gli2*); Egf pathway (*Hb-egf*, *Egfr*); hypoxia pathway (*Hif1a*, *Vegfa*, *Pdgfb*). Each symbol shows data from one mouse. Mean ± SD. Two-way ANOVA with Tukey's multiple comparisons test. * P value <0.05; ** P value <0.01.

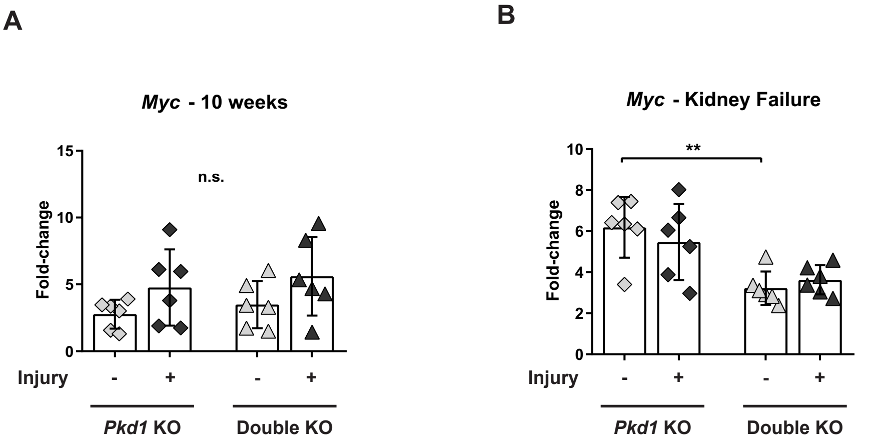

Figure S4. Wnt pathway target Myc (A) Gene expression of *Myc* 10 weeks after injection of the nephrotoxic compound S-(1,2-dichlorovinyl)-L-cysteine (DCVC) or PBS in *Pkd1* KO mice and *Pkd1*/*Fjx1* double KO mice. (B) Gene expression of *Myc* at kidney failure in *Pkd1* KO mice and *Pkd1*/*Fjx1* double KO mice. Each symbol shows data from one mouse. Mean ± SD. Two-way ANOVA with Tukey's multiple comparisons test. ** P value <0.01.

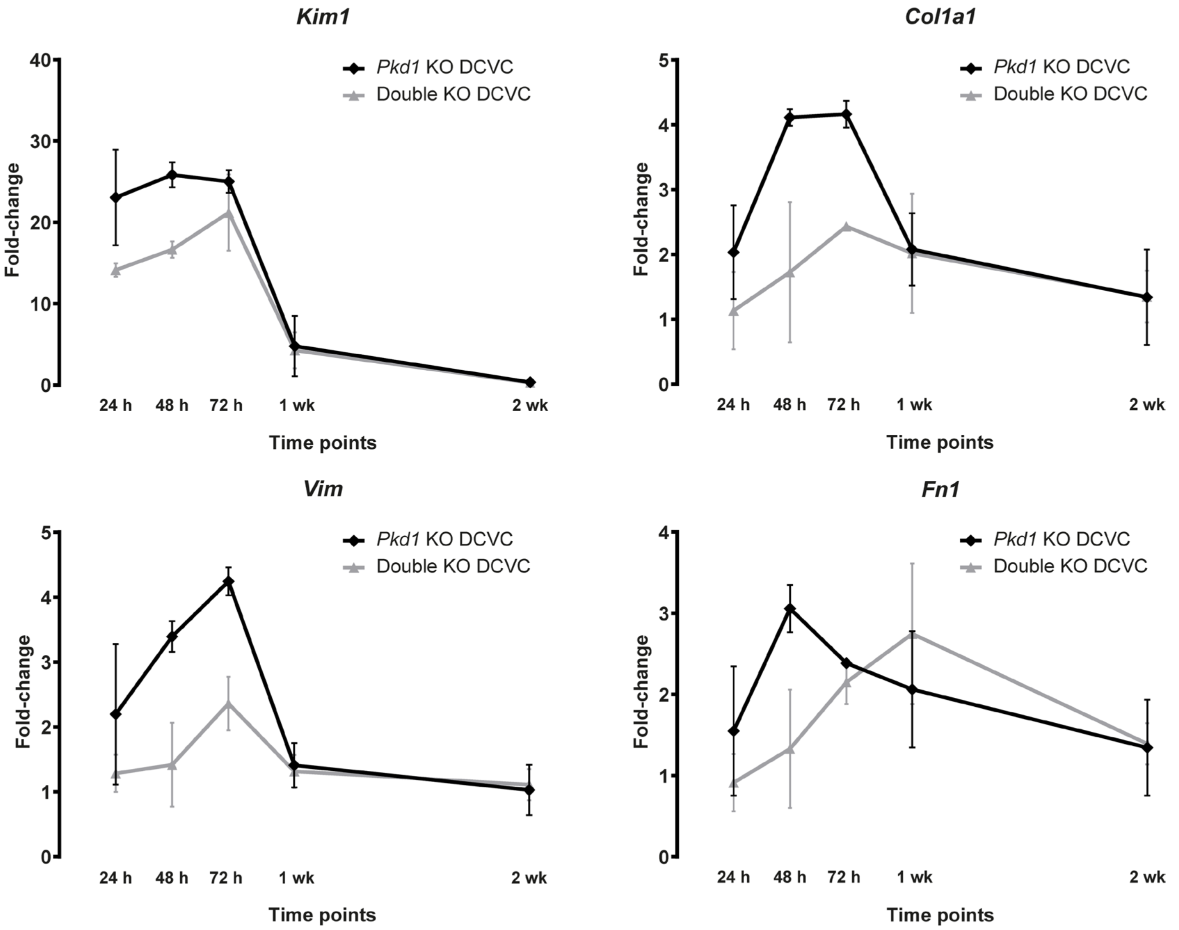

Figure S5. Injury and fibrotic genes expression at early time points after injury. Gene expression of *Kim1*, *Col1a1*, *Vim* and *Fn1* at 24 h, 48 h, 72 h, 1 and 2 weeks after S-(1,2-dichlorovinyl)-L-cysteine (DCVC) injection. Each point is the mean of two mice (24 h, 48 h, 72 h) or six mice (1 and 2 weeks) ± SD.

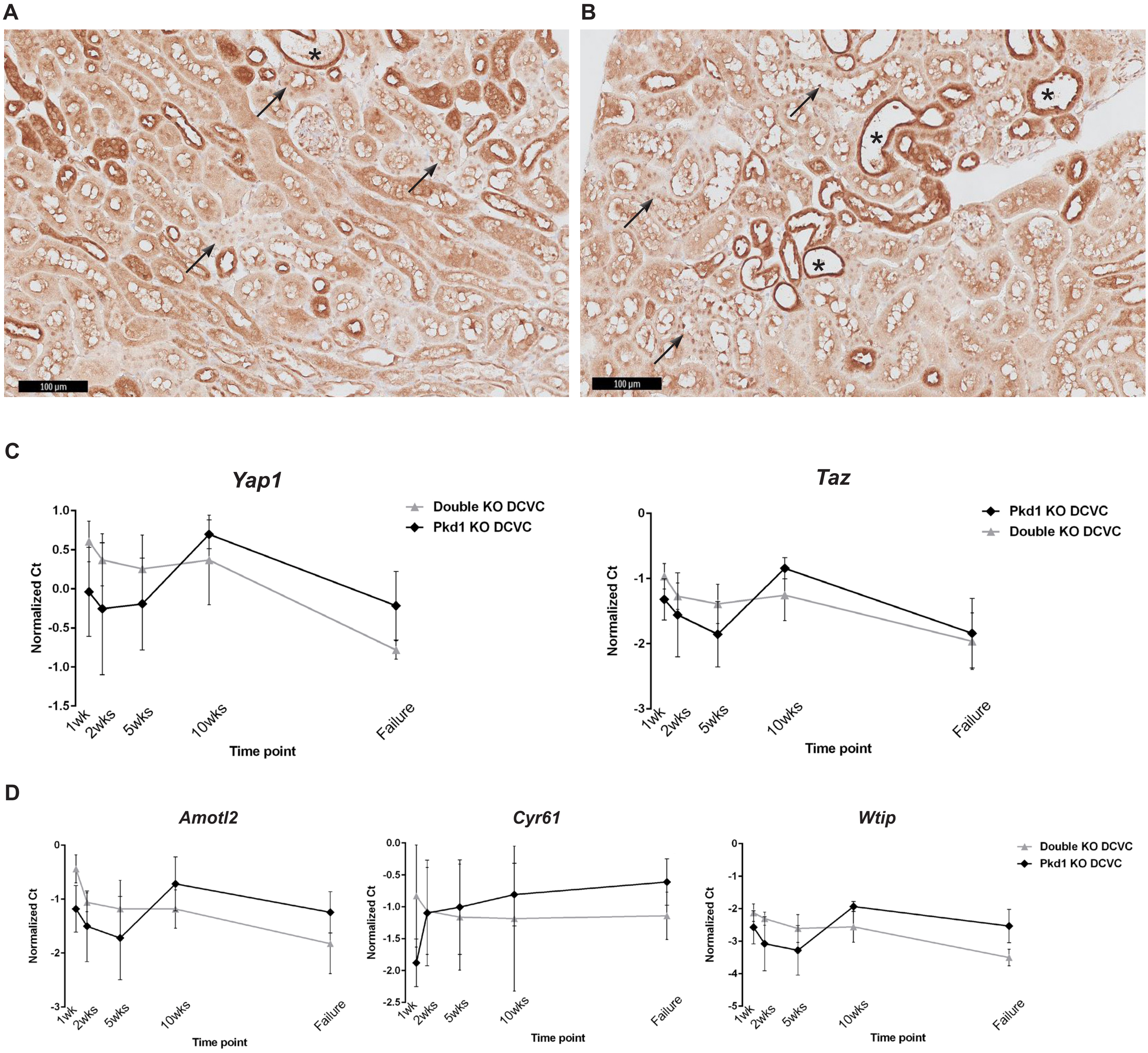

Figure S6. Hippo Pathway activation in Pkd1 KO and Pkd1/Fjx1 double KO mice. (A) Representative immunostaining for Yap1 on kidney tissue in *Pkd1* KO mice after injection of the nephrotoxic compound S-(1,2-dichlorovinyl)-L-cysteine (DCVC) and (B) double KO mice after DCVC. In both genotypes, it is possible to observe some dilated tubules which intensely stain for Yap1 and diffuse nuclear localization. Scale bars, 100 μm. Arrows indicate tubules showing nuclear Yap1; asterisks indicate dilated tubules. (C) *Yap1* and *Taz* expression during disease progression. (D) Gene expression of representative Yap1/Taz targets. Each point is the mean expression of six mice ± SD.
