# Supplementary material for: Four‐jointed knock‐out delays renal failure in an ADPKD model with kidney injury
Source: J Pathol. 2019 Jun 17;249(1):114–25. doi: 10.1002/path.5286 (PMC6772084; doi:10.1002/path.5286)
Supplement: Supplementary file 3 — Table S1. List of mouse qPCR primer sets used [file PATH-249-114-s002.docx]

**Four-jointed knock-out delays renal failure in an ADPKD model with kidney injury**

Formica C *et al*. *J Pathol* DOI: 10.1002/path.5286

| **Table S1.** List of mouse qPCR primer sets used. | | |
| --- | --- | --- |
|  |  |  |
| Target | **Forward** | **Reverse** |
| **Kim1** | TCTCTAAGCGTGGTTGCCTT | TGTCTTCAGCTCGGGAATGC |
| **Col1a1** | TGACTGGAAGAGCGGAGAGT | AGACGGCTGAGTAGGGAACA |
| **Vim** | CCAACCTTTTCTTCCCTGAA | TGAGTGGGTGTCAACCAGAG |
| **Fn1** | AATCCAGTCCACAGCCATTCC | CCTGTCTTCTCTTTCGGGTTCA |
| **aSMA** | CATCATGCGTCTGGACTTG | ATCTCACGCTCGGCAGTAG |
| **TGFb** | ACTATTGCTTCAGCTCCACAGA | AAGTTGGCATGGTAGCCCTT |
| **F4/80** | GGCAGGGATCTTGGTTATGCT | GCTGCACTCTGTAAGGACACT |
| **Fat4** | ACCGATGCAGATGATGGTGTC | ACTCCGTGCTTATCCACTGC |
| **Dchs1** | GACAATCGTCCCACCATCCC | AGCCCAACAGTGCATCTTCT |
| **Yap1** | TTCCGATCCCTTTCTTAACAGT | GAGGGATGCTGTAGCTGCTC |
| **Taz (Wwtr1)** | ATGGACGAGATGGATACAGGTGA | AGACTCCAAAGTCCCGAGGT |
| **AmotL2** | ACCAGGAGATGGAGAGCAGATT | GAAGGACCTTGATCACCGCA |
| **Cyr61** | CACTGAAGAGGCTTCCTGTCT | CCAAGACGTGGTCTGAACGA |
| **Wtip** | TTCATCTGTGACTCCTGTGGGA | TGGCAGTACACTTTCTCACCC |
| **Notch1** | GGTGCTCTGATGGACGACAA | TACTGGCTCCTCAAACCGGA |
| **Notch2** | AGGCTAACCTGATTGGTTCTGG | AAGCCTCATCCTCAGCCTTG |
| **Notch3** | CTGGGAGTCAGTGTCAGAACC | GGTGGACAAATGCAGTAAGCC |
| **Hes1** | GGCCTCTGAGCACAGAAAGT | TTGGAATGCCGGGAGCTATC |
| **HeyL** | AAGAAGCGCAGAGGGATCATAG | GGGACCAATCGTCGCAATTC |
| **Gli1** | CAGCATGGGAACAGAAGGACT | ACCCTGGGACCCTGACATAA |
| **Hb-EGF** | GAGGAGGACCTGAGCTATAGGAA | AACGTGTAACGAACCACTGTCT |
| **EGFR** | GAAGTACAGCTTTGGTGCCACCTG | CTTGCGGATGCCATCTTCTTCCAC |
| **HIF1a** | AGTCAGCAACGTGGAAGGTG | GCACGTCATGGGTGGTTTCT |
| **VEGFa** | CTCCACCATGCCAAGTGGTC | GTCCACCAGGGTCTCAATCG |
| **PDGFb** | CAAGAGTGTGGGCAGGGTTAT | CCGAATCAGGCATCGAGACA |
| **Axin2** | GACAGCGAGTTATCCAGCGA | AGGAGGGACTCCATCTACGC |
| **Jak2** | ACCTTTGCTGTTGAGCGAGA | CTTAGTCCCGCTGAGGTTGT |
| **Stat1** | TTCCGACACCTGCAACTGAAG | TCTTCGGTGACAATGAGAGGC |
